# Radiation cataracts: mechanisms involved in their long delayed occurrence but then rapid progression

**Published:** 2008-02-05

**Authors:** Norman Wolf, William Pendergrass, Narendra Singh, Karen Swisshelm, Jeffrey Schwartz

**Affiliations:** 1 Department of Pathology; 2 Department of Bioengineering, and; 3 Department of Radiology, University of Washington, Seattle WA

## Abstract

**Purpose:**

This study was directed to assess the DNA damage and DNA repair response to X-ray inflicted lens oxidative damage and to investigate the subsequent changes in lens epithelial cell (LEC) behavior in vivo that led to long delayed but then rapidly developing cataracts.

**Methods:**

Two-month-old C57Bl/6 female mice received 11 Grays (Gy) of soft x-irradiation to the head only. The animals’ eyes were examined for cataract status in 30 day intervals by slit lamp over an 11 month period post-irradiation. LEC migration, DNA fragment, free DNA retention, and reactive oxygen species (ROS) presence were established in the living lenses with fluorescent dyes using laser scanning confocal microscopy (LSCM). The extent and removal of initial LEC DNA damage were determined by comet assay. Immunohistochemistry was used to determine the presence of oxidized DNA and the response of a DNA repair protein in the lenses.

**Results:**

This treatment resulted in advanced cortical cataracts that developed 5–11 months post-irradiation but then appeared suddenly within a 30 day period. The initially incurred DNA strand breaks were repaired within 30 min, but DNA damage remained as shown 72 h post-irradiation by the presence of the DNA adduct, 8-hydroxyguanosine (8-OHG), and a DNA repair protein, XRCC1. This was followed months later by abnormal behavior by LEC descendant cells with abnormal differentiation and migration patterns as seen with LSCM and fluorescent dyes.

**Conclusions:**

The sudden development of cortical cataracts several months post-irradiation coupled with the above findings suggests an accumulation of damaged descendants from the initially x-irradiated LECs. As these cells migrate abnormally and leave acellular lens surface sites, eventually a crisis point may arrive for lens entry of environmental O_2_ with resultant ROS formation that overwhelms protection by resident antioxidant enzymes and results in the coagulation of lens proteins. The events seen in this study indicate the retention and transmission of progenitor cell DNA damage in descendant LEC. The cellular and molecular events parallel those previously reported for LSCM observations in age-related cataracts.

## Introduction

The in vivo behavior of functional cells and their descendents after receiving oxidative damage from reactive oxygen species (ROS) is of major biologic importance. Equally important are the agents and mechanisms involved in producing that damage as well as its cell and tissue signature and its repair. We have explored the sequence of these events in a single layer of lens epithelial cells (LECs) on the surface of the front half of the mouse lens that migrate and differentiate to become the internal lens fiber cells and then the lens fibers. For the source of oxidative damage, we have used soft X-rays. The literature contains ample evidence that several types of radiation, such as heavy charged particles [1,2], neutrons [3,4-6], gamma radiation [4,6,7], X-ray [8-12], ultraviolet [13,14,15], microwaves [9], and even white light [16], produce oxidative damage in the lenses of several mammalian species, often leading to the development of cataract. We have undertaken a study of the effects of a single dose of soft, low energy x-irradiation (5 mA, 100 kV) delivered only to the head of two-month-old mice in which the primary damage inflicted on LEC is due to the formation of ROS rather than direct energy transfer [17-21]. One reason for this approach was to compare this form of oxidative damage to the lens and the expected induction of cortical cataracts to that of age-related cataract (ARC), a condition that we and others have shown to be at least in part due to LEC damage inflicted over time from ongoing oxidative events of biologic origin [11,22-27]. Using dual laser scanning confocal microscopy (LSCM) assessment of fluorescent dyes specific for DNA presence and for ROS, we found that the failure of LEC differentiation and their abnormal migration from the lens surface at inappropriate sites accompanied by localized ROS all of which were previously demonstrated in ARC [28,29] were present in the X-ray induced cataracts. However, unlike the gradual development of ARC, X-ray induced cataracts developed suddenly after a 5–11 month delay following irradiation. At that time, the X-ray induced cataracts became visible by slit lamp and developed within a 30 day period in the then 7- to 13-month-old mice, an age at which non-irradiated controls remained free of cataracts. The very delayed yet sudden development of these cataracts eliminates the possibility that they are caused by changes in the internal lens proteins at the time of irradiation. This allows us to focus on the post-irradiation behavior of LEC and its relationship to cellular DNA damage. In these studies, we show that the progenitor LEC survive the radiation but bear DNA damage requiring efforts at repair and exhibit abnormal functional behavior that may explain the late development of cataracts.

## Methods

### Animals and tissues

The first set of 12 irradiated and 12 control C57BL/6 female mice were two months old at the time of the primary radiation experiment and were followed for timing and extent of cataract development by monthly slit lamp examinations for 11 months after the irradiation. A second group of 12 irradiated plus 12 control animals that were also two months of age, similarly irradiated, and of same sex and strain was used to provide irradiated and control lenses, respectively, for cell culture of LEC, half for clonal studies and half for metaphase spreads. A third group of such mice were used for antibody studies of 8-hydroxyguanosine (8-OHG) and DNA repair antibody presence 72 h post-irradiation. Lenses were isolated by posterior excision from the extirpated eyeball 2 min after euthanasia of the mice by cervical dislocation. Only females were used to avoid the stress and fighting damage often seen in separately weaned and caged males of this strain. These specific pathogen-free animals were purchased from Harlan Laboratories, Inc (Indianapolis, IN) and housed four per cage and fed the standard maintenance Purina mouse diet 5015 (Purina Labs, St. Louis, MO). Humane protocols for maintenance and euthanasia from the University of Washington IACUC committee and the national AALAC organization were followed.

### Lens treatments

When used for the comet assay, the quickly extirpated lenses were placed immediately over ice to prevent enzymatic activity. Lenses for immunohistochemistry were immediately placed in Carnoy’s fixative. Lenses for confocal examination were placed sequentially in the Hoechst 33342 and the dihydrorhodamine dyes, which were diluted in Eagle’s minimal essential medium (MEM) immediately after removal, with the total period in these dyes not exceeding 2 h. Controls in which the dyes were followed from the first to the fourth hour showed no change in the dye penetration or in the final confocal readout. Further, the differences seen in dye presence between the previously irradiated lenses and the non-irradiated control lenses did not change over the length of time that they were allowed to remain in the dye solution.

### Radiation source, dosage, and effects

A cabinet X-ray machine (Picker Company, Cleveland, OH; model 43855A, Series 209, cycles 50/60, Pre volts 115 with PKV 110 and Beryllium window) was used to irradiate mice placed 13 inches from the source. Delivery of 11 Grays (Gy) of X-rays was at 5 mA, 100 kV over 5.5 min. This is a low energy delivery compared to energy delivered by a 300 kV therapy machine or a Cesium source. Five mice at a time were placed on a circular rotating base with heads to the center and the remainder of the body including the tail covered with 3 mm of lead. Thus, only the heads were exposed to the x-radiation. The settings and delivery were measured by specialists from the University of Washington Environmental Health Division using a Victoreen meter. The mice were anesthetized with a mixture of ketamine (100 mg/ml) and xylazine (20 mg/ml) diluted with saline 12.6 fold to deliver anesthesia at 0.02 ml/gm of bodyweight. This was injected intraperitoneally (I.P.). just before the irradiation. They remained anesthetized for approximately 30 min. None of these animals in either the control or the head-irradiated groups showed any signs of distress, reduced physical activity, or reduced food intake during the study period from the beginning to the conclusion of the experiment 11 months following the irradiation. There was a general hair loss from the head that was obvious two weeks after irradiation with re-growth of melanin-free hair over the subsequent three to four weeks. Two mice developed sufficient subsequent scar tissue near one eye in each animal that while not covering the eye, the scar tissue prevented an accurate slit lamp reading of cataract development on that side of the face.

### Cataract examinations in the living animals

The irradiated mice and non-irradiated controls in group 1 were examined every 30 days through the 11^th^ month post-irradiation by slit lamp using a Kowa SL14 hand held slit lamp (Kowa, Tokyo, Japan) after dilation with a 3:1 volume mixture, respectively, of tropicanamide (Acorn, Buffalo Grove, IL) and phenyl hydrochloride (Acorn, Buffalo Grove, IL), human use ophthalmic solutions that achieved full dilation. The viewer was blinded as to the animal’s previous treatment. The degree of lens opacity was rated by half steps from 0 (completely clear) to 4 (complete opacity of a mature cataract). When lenses were removed from the euthanized animals for the confocal studies, they were first scored for opacity using a dissecting microscope. Thus, in each sacrifice of two mice each in the X-ray and the control groups at each time point at one, three, five, and seven months, the slit lamp cataract readings increased in severity in the irradiated mice as noted in the Results section. The surgically removed lenses were immediately examined under a dissecting microscope and rated separately by two persons without knowledge of the irradiated or control status of the donor mouse. There was no more than a 0.5 difference on the 0 to 4 point rating scale between the live animal slit lamp scoring and that made under a dissecting microscope for those lenses removed surgically for confocal study (notations of 1+ to 4+ indicate that the rating level had reached that stage in either type of examination). Confocal, fluorescent dye readings were done immediately after the dissecting microscope reading. These confocal readings rated the corresponding statuses of the LEC nuclear DNA clearance, the internal lens DNA distribution (both fragmented and in free form), and the ROS presence. These, in turn, corresponded with the live animal slit lamp and the post-sacrifice dissecting microscope readings for increasing lens opacity with all three factors increasing with time beginning at five months post-irradiation. It is noted that the appearance of the individual cataracts increased over time but was nevertheless sporadic with some not appearing until the seventh month or beyond. Thus, the selection of donors for confocal studies was made from among those mice presenting with cataracts as determined by slit lamp examination. However, as no cataracts were present at one month and three months post-irradiation, random animals from each group were selected for confocal study at these times.

**Table 1:** Metaphase cells with one or more abnormal chromosomes

Animal status and age	Total number of abnormal chromosomes (abnormal cells/total cells in metaphase)	Types of aberrations
Eight-week-old control	15 (2/13)	Small marker chromosomes
Eight-week-old irradiation	27 (4/15)	Chromatid break; trisomy 8; trisomy 18; + marker chromosomes
26-month-old control*	0 (0/5)	
26-month-old radiation*	30 (3/10)	+ small markers; chromatin unraveling

The asterisk indicates that a set of 26-month-old animals has been added from a separate but same procedure X-ray study to increase the total number of cells examined for post X-ray chromosomal abnormalities. The first number indicates the total number of abnormal chromosomes found. The numbers inside the parentheses indicate the fraction of abnormal cells (some with several abnormal chromosomes) over the total cells that were present in a complete metaphase spread condition.

### Sacrifice times for confocal studies of living whole lens and for Carnoy’s-fixed paraffin-sectioned, antibody studies

Groupings of two mice each from the irradiated group and their non-irradiated controls were sacrificed at three, five, and seven months post-irradiation for living lens collections and for immediate exposure to the fluorescent dyes listed below followed by dual laser confocal examination. Sacrifices 72 h post-irradiation were from the third group of mice and were fixed in Carnoy’s fixative and used to prepare 10 μM lens tissue sections using fluorescence antibody studies of the DNA adduct, 8-OHG, and the DNA repair protein, XRCC1. Sacrifice of mice for LEC in vitro culture studies was at 72 h post-irradiation followed by culture as noted below. Mice were first anesthetized then sacrificed by cervical dislocation.

**Figure 1:**
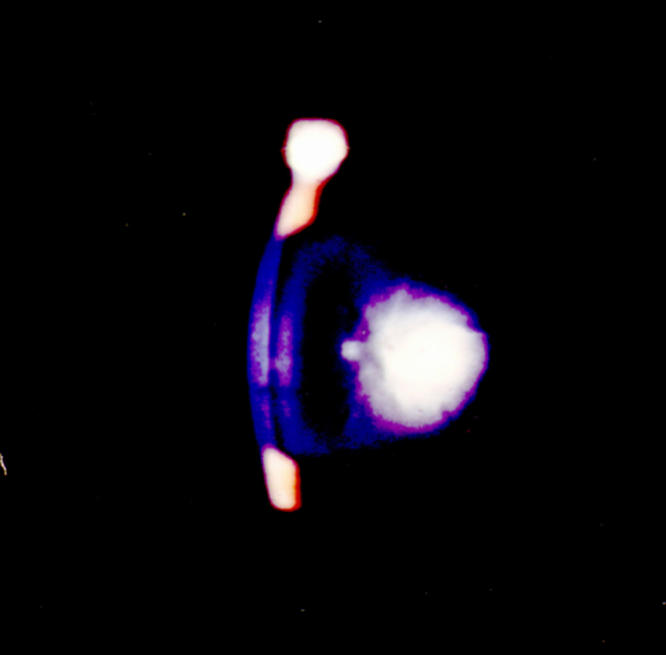
A level 4 cataract as viewed by slit lamp seven months post-irradiation. This cataract developed over a period of 30 days at seven months post-irradiation. It was not present at six months, and the same lens was scored as 1.5+ at that time. The same minimal degree of opacity (1–2+) was also seen in the non-irradiated control mice at that age.

### Comet assay for DNA strand breaks

The integrated comet alkaline gel migration assay as described in Singh et al. [16] integrates the values for length, breadth, and intensity of the DNA stained comet tail. The alkaline gel conditions report single strand breaks and alkaline sensitive sites that allow breaks to occur. It was run on the LEC freed from the eight lenses of four mice per each preparation at 5 min, 15 min, 30 min, and 4 h post-irradiation. Removal of the lenses required 2 min. The lenses were placed in small tubes immersed in ice immediately upon extirpation to stop enzyme activity. The LEC were obtained from the lens within 1 min by dispersal in a device as previously published [16].

### Use of fluorescent dyes and dual laser confocal microscope scanning of whole living lenses

Briefly, a computer-assisted Zeiss 2-photon LSCM (model 510 NLO, Carl Ziess, MicroImaging Inc., Thornwood, NY) was used on whole lenses immediately after extraction and subsequent 60 min immersion in MEM media containing 10 uM Hoechst 33342 dye for DNA detection and quantitation and 25 uM dihydrorhodamine (DHR) for ROS detection and quantitation. For LSCM settings and dye dilutions see Pendergrass et al. [29]. The source of both dyes was Molecular Probes Inc. (Eugene, OR). Only one fluorochrome was excited at a time to assure that only the desired probe was visualized. Analyses were performed at both the anterior and posterior poles of the lenses using a 10X objective and scanning 31 frames from an area of the lens 1.3 mm in diameter and to a depth of 120 μm deep into the cortex. Deeper scans were collated at a succession of 8 μm depths in a staggered series from the lens surface to a maximal depth of 120 μm and were made to assess the extent of the regions affected. These were also extended to determine whether cataracts extended into the nucleus of the lens, which the larger cataracts often did. For assurance concerning the presence of DNA in both nuclear fragments and free DNA in the lens cortex, some paired sections were first fixed with 5% paraformaldehyde and then treated with DNase. These showed complete removal of the Hoechst stained entities. From the first group of 12 irradiated plus 12 control mice, two mice from each group were sacrificed at three, five, and seven months post-irradiation for the confocal lens examinations. Additional LSCM settings and procedures information is available in references, [28,29].

**Figure 2:**
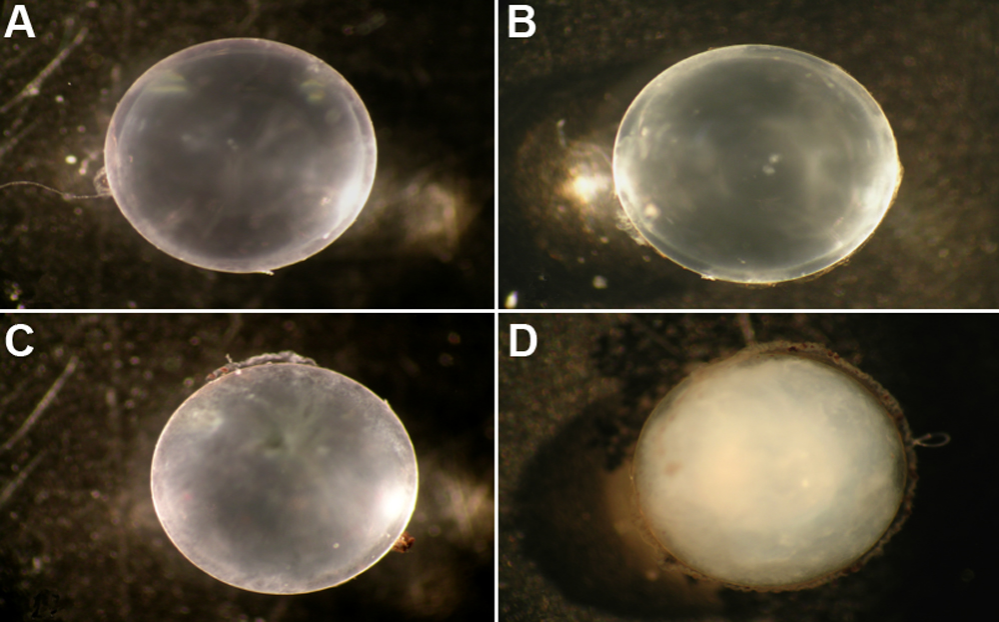
Typical lenses removed and viewed by 10X microscopy. **A:** This panel shows a non-irradiated control mouse five months after initiation of the experiment with lens opacity blinded slit lamp rating of 1+. **B**: Shown here is the lens of an irradiated mouse one month after irradiation. There is minimal, if any, opacity present. **C**: Shown here is an irradiated mouse lens three months after irradiation, and rated 1.5+. **D**: Shown is a fully developed cataract rated 4+ that developed this advanced opacity within a 30 day period five months after irradiation. Only two of the irradiated mice developed cataracts at five months with the remainder of the mice developing cataracts between 7 and 11 months post-irradiation and ,in each instance, within a 30 day period.

### Antibody staining techniques

All antibody staining was by primary and secondary antibody technique of Carnoy’s-fixed, paraffin-embedded serial sections with viewing and photography using a Bio-Rad (Hercules, CA) computerized microscope with camera attachment. The antibody used for 8-hydroxyguanosine staining (8-OHG; Alpha Diagnostics Inc., San Antonio, TX) was rabbit polyclonal anti-mouse. The primary antibody used for XRCC1 staining was also rabbit polyclonal anti-mouse from the same source. All secondary antibodies were fluoresceinated goat-anti rabbit, which was also attained from the same source. Control slides substituted non-specific rabbit IgG for the primary antibody also from Alpha Diagnostics. Paraffin block preparations fixed in Carnoy’s fixative were found to be superior for sections and antibody stains when compared to frozen sections. Coomassie blue dye was used on a Carnoy’s-fixed tissue section to illustrate the aggregated protein status in a 4+ cataract.

### Chromosome damage studies

From the second similarly irradiated group, noted above, the lenses from six irradiated mice and an equal number of non-irradiated controls were removed 72 h post-irradiation; the LEC were collected by stripping them as a layer from the surface of the lens with forceps and placing them in a 1% collagenase/dispase and 3% bovine serum albumin (BSA) solution for 30 min at 37 °C. Completion of LEC separation was accomplished with forceps followed by collagenase/dispase dispersal. A control group consisted of mice of the same age and sex that did not receive the irradiation but were otherwise treated similarly. The LEC were then passed through a 70 μm filter, plated in DMEM+10% fetal calf serum, and cultured for a total of 12 days in 60 mm plates (Biocoat; Becton Dickenson, San Jose, CA) into which glass coverslips has been placed. Metaphase spreads were made from cultured LEC of irradiated mouse lenses removed 72 h post-irradiation and cultured for a total of 12 days under 3% O_2_. Twenty-four h before fixation in Carnoy’s fixative, the cells growing on the coverslips were exposed to 0.01 µg/ml colchicine overnight to accumulate metaphases. The coverslips were mounted on glass slides and the resulting metaphase spreads that were treated by Wright-trypsin G-banding procedures were read by one of us (K.S.) for chromosomal aberrations. To increase the number of metaphase cells scored, we have included in Table 1 a small number from old mice that were treated in the same manner as the young mice. This is the only entry for old mice in this study.

**Figure 3:**
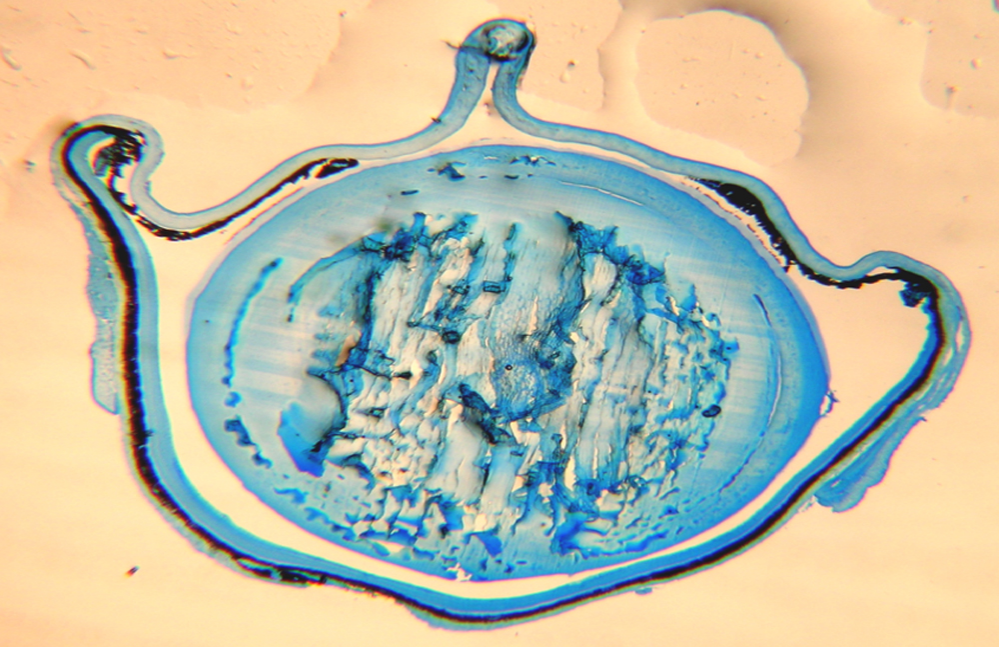
Aggregated proteins are prominent in the nucleus and inner cortex of the severely cataractous lens. A Comassie Blue protein stained lens section from an extensive post-irradiation cataract shows aggregated protein at the quite extensive 4+ cataract site.

### Clonal lens epithelial cell growth in vitro

LEC from six animals, each collected from the irradiated and the non-irradiated subgroups of the second group of irradiated and control mice, as noted above, were separately pooled after sacrifice of the mice at 72 h post-irradiation. The cells were collected and dispersed using collagenase/dispace and grown in an MEM medium containing 10% fetal bovine serum (FBS). After seven days in the original mass plating, they were then freed with collagenase/dispase, washed in phosphate buffered saline (PBS), and singly and sparsely replated in triplicate in a similar medium on 35 mm collagen coated plates for individual cell clonal growth. After nine days in the second plating, the size of the individual clones formed was assayed by counting individual cells in each clone after crystal violet staining. The clone counts were then assayed for the percent of clones in large or small cell content groups using several size classification sets (1–4 cells, 30–50 cells, 50–75 cells, >100 cells per clone). Several combinations of these clone number classification groups were used to determine whether significant differences in the percentage of the present large or small clones existed between the collections for the irradiated donors and the non-irradiated controls. Total numbers of clones of any size were also recorded.

### Statistical analysis

The degree of DNA presence and DHR in the cortex was compared between irradiated and control lenses for fluorescence intensity by confocal readings from several lens depths using the Mann–Whitney non-parametric test.

**Figure 4:**
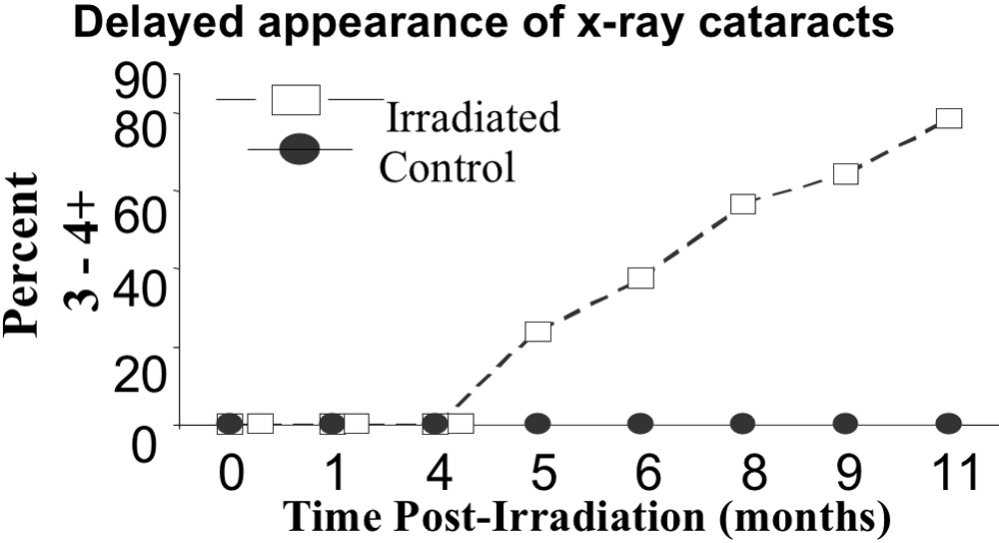
X-ray cataracts as assessed by slit lamp appeared suddenly after several months delay. Shown here is the percent of cataractous eyes present in the irradiated and the control groups at monthly examinations following the irradiation. Ordinate: percent of lenses so affected. Abscissa: time post-irradiation in months.

## Results

### Delayed but rapid appearance of X-ray induced cataracts

The typical cataracts that appeared over a 30 day period but only 5-11 months after radiation are shown in Figure 1 as a level 4 cataract that changed from 1.5+ to 4+ opacity within 30 days at seven months post-irradiation when examined by slit lamp in vivo. In Figure 2, the typical lens status is shown immediately after lens removal at one, three, and five months post–irradiation. Lens sections from cataractous lenses stained with the protein dye Coomassie Blue revealed extensive protein aggregation (Figure 3). The status of all lenses of both the treated and control animals remained at minimal opacity (0–0.5+ rating on a 4 point scale) early after irradiation and then progressed only modestly (to a maximum of 2+) with advancing age at each 30 day interval reading until the fifth post-irradiation month. At that time, complete opacity (3.5−4+) suddenly appeared in the first two individual eyes of two individual irradiated mice. In the following months, there were additional random appearances sometimes in one eye and sometimes in both eyes. The accumulation of 3.5–4+ lens readings is shown for individual eyes in Figure 4. By the 11^th^ month post-irradiation, 80% of the irradiated eyes were positive for advanced cataract at which time the experiment was ended. In all instances and at all observational time points, the individual 3.5–4+ cataracts developed within the 30-day observation periods from previous readings of 1.5–2+. Only opacities scored as 3.5+ to 4+ were considered to be cataracts with 3.5+ representing an advanced opacity including the entire lens as viewed by slip lamp and 4+ representing a lens opacity with 100% light reflection (complete opalescence). As seen in Figure 2 and Figure 4, the non-irradiated control mice changed their lens opacities minimally and gradually, only reaching levels 1.5–2+ by the end of the experiment, at which time all of the mice were 13 months old. No abnormalities of LEC migration, DNA, or ROS presence in confocal studies were seen in the controls that accompanied the irradiated mice at each sacrifice time.

**Figure 5:**
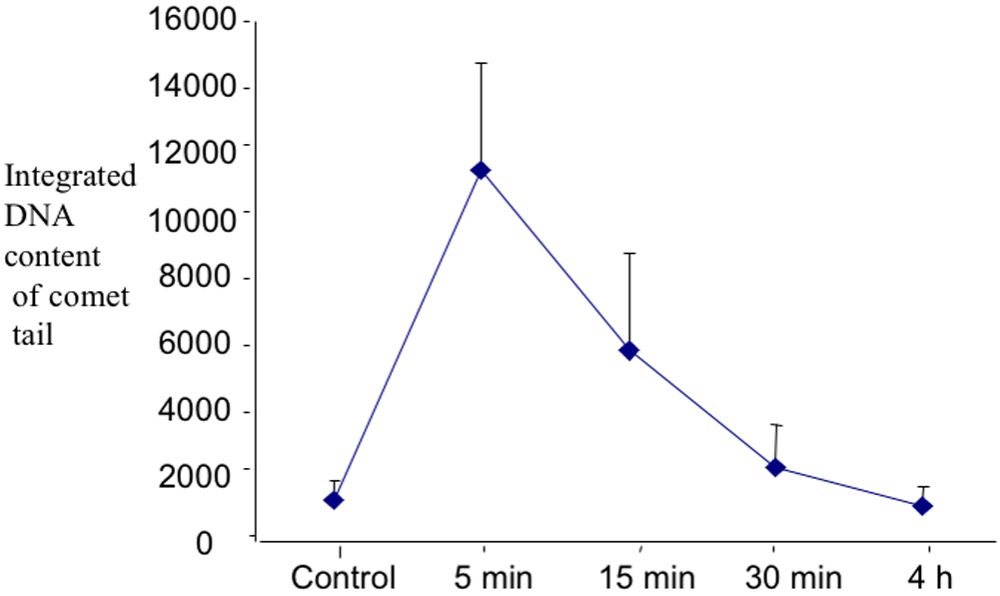
Time scale of comet measurement of DNA fragment presence by alkaline integrated comet assay. This assay integrates the content of migrating DNA fragments at a progressive distance in the gel where the extreme margin is the tip of the comet tail, i.e., integrating the DNA fragment content per linear distance, with the furthest progression through the gel. Means and SEM are shown.

### Recovery from the initial radiation-induced DNA breakage

The comet assay measures primarily single strand breaks and alkali labile sites if run under alkaline conditions as it was here [16]. Although the LEC from lenses of the mice sacrificed at 5 min post-irradiation showed large amounts of DNA breaks, essentially complete recovery was apparent in lenses taken 30 min post-irradiation (Figure 5).

**Figure 6:**
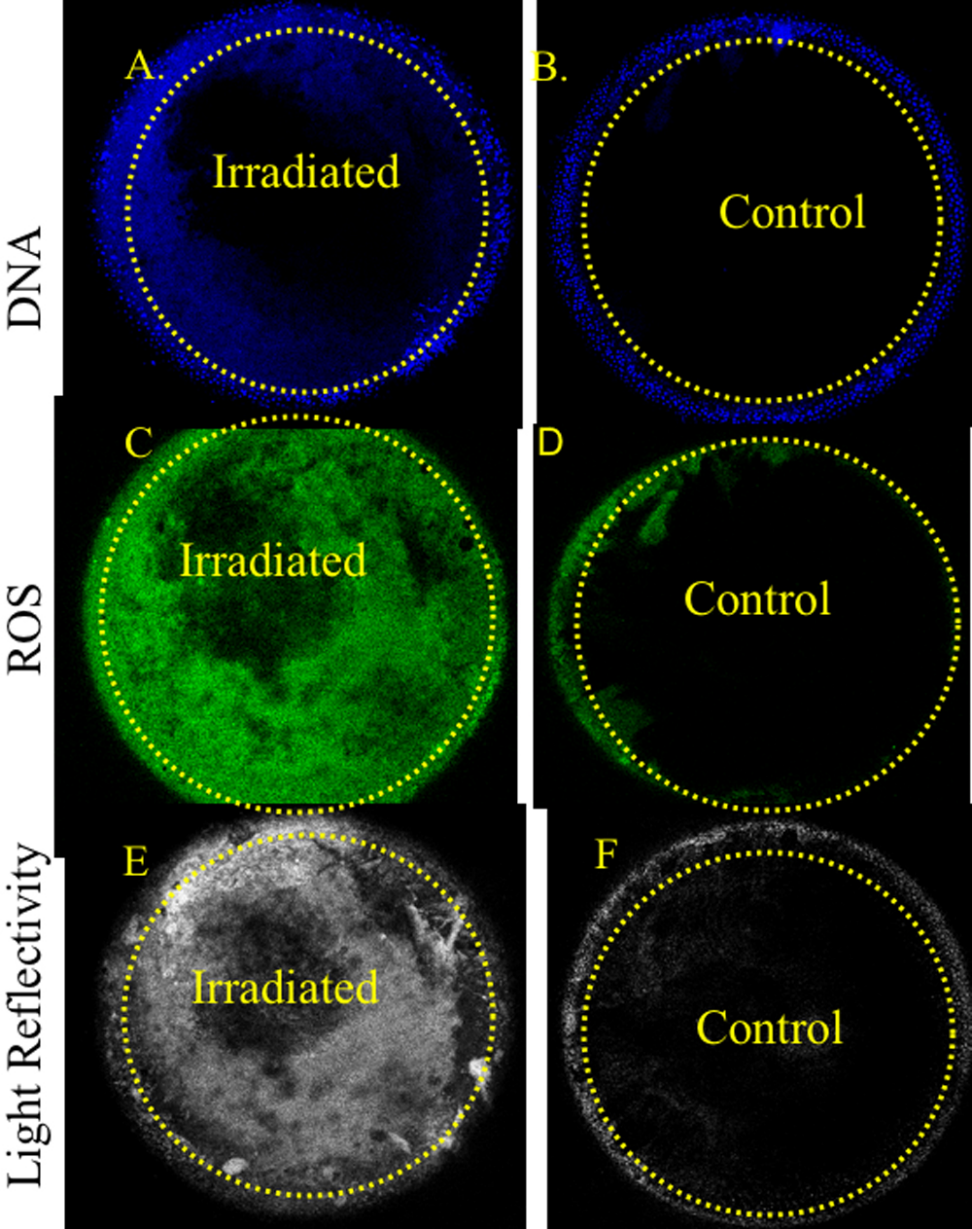
The presence of free DNA and ROS in the x-ray cataract. Using LSCM with fluorescent dyes for DNA and ROS, integrated sequential sections from the surface down to 120 μm beneath the lens surface revealed the following in a 3.5+ x-ray cataract that developed 5 months post-radiation: **A**: Free and particulate DNA is present in the left upper part of the irradiated lens. **B**: A control animal at the same age and time is free of the abnormal internal DNA content. **C**: A great amount of internal ROS is present in the irradiated lens at several depths. **D**: The control animal’s lens shows a very small amount of ROS near the lens surface. The dotted lines were placed on the lens sections to demarcate the position of the lens surface.

**Figure 7:**
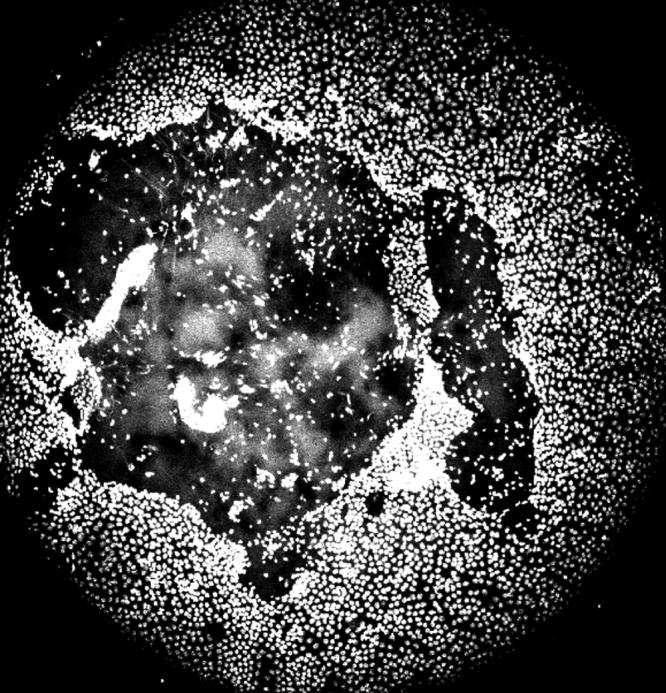
Loss of surface LEC by death and by abnormal migration in the x-ray cataract. This confocal view shows the loss of surface LEC and their descent into the lens interior at a site well anterior to the lens equator. The irradiated animal is five months post-irradiation. This picture is made from “stacked” multiple images extending from the lens surface to 120 μm into the interior.

In spite of the initial recovery of DNA strand breaks, a dysfunctional lens status increased over time as confirmed by confocal viewing of living lenses using fluorescent dyes for nuclear fragments, free DNA presence, and localized ROS presence as well as noting aberrant LEC migration within the lens. These changes increased progressively in the irradiated mice when viewed in this manner. They were not present in the first month but developed minimally by the third month and were present as marked changes in cataractous, irradiated lenses at five and seven months post-irradiation (Figure 6) with aberrant LEC migration (Figure 7). Thus, the slit lamp and confocal microscopic sequential examinations were usually in agreement, although the confocal examinations at three months showed some minimal early loss of surface LEC while the slit lamp examinations did not yet reveal any evidence of cataract. We selected only those pairs of irradiated mice with obvious opacities when observed by slit lamp examination and pairs of control mice for the confocal studies at five and seven months post-irradiation to illustrate and measure by degree of respective dye fluorescence the internal and surface LEC lens changes. Thus, the most likely candidates were chosen from the slit lamp results for use in the confocal examinations. The advanced changes in a cataractous mouse lens at five months post-irradiation (shown in the confocal view in Figure 6) displayed Hoechst 33343 fluorescence for displaced DNA presence, dihydrorhodamine conversion to rhodamine for ROS presence, and light refraction at the affected sites (note the spatial overlap for the each of these). Both the free DNA and the ROS computerized fluorescence measurements differed from those of control mice by p<0.01 at five months post-irradiation and p<0.001 at seven months using the Mann–Whitney non-parametric test. These measurements included all regions of the interior lens as “stacked” confocal readings of 6 μm each to a 120 μm depth in the cortex. The overall cortex readings contained an accumulation of both nuclear fragments and free DNA. In general, the ROS intensity was greatest at internal lens regions where DNA intensity was also highest. Sequential depth confocal views also showed the migration of LEC strands to the lens interior from inappropriate surface sites that were not at the normal subequatorial bow region. This misdirected migration was a prominent feature in the cataractous lens, and the internalized strands of cells present there did not undergo resolution of their nuclei as shown in the stacked depths integrated LSCM readings in Figure 7. Each of the above noted events are quite similar to those seen in age-related cataract in non-irradiated old mice of the same strain and in old rats as we have previously reported [28,29].

**Figure 8:**
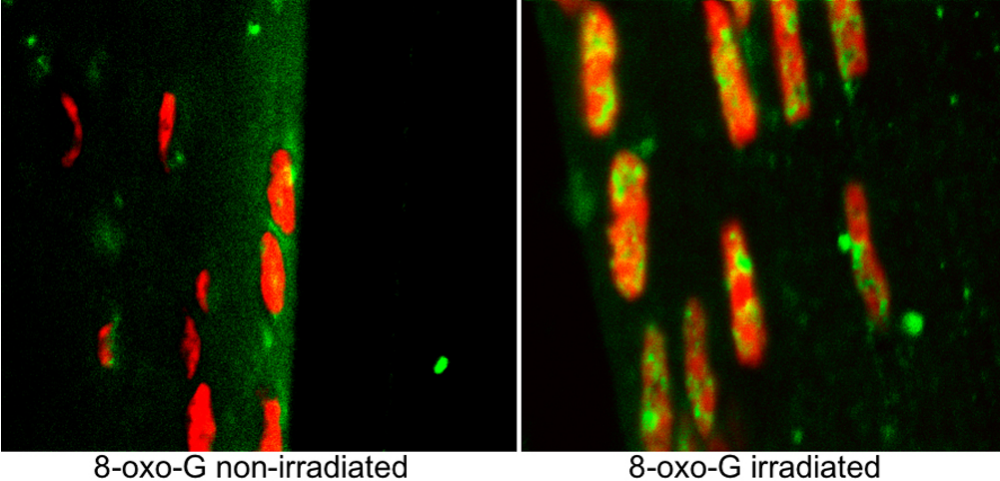
The DNA adduct 8-OHG is present in surface LEC nuclei in the x-ray cataract. Direct antibody for 8-OHG was applied followed by secondary fluorescent antibody staining in a Carnoy’s fixed paraffin section. The non-irradiated animal’s lens is on the left and the irradiated is on the right, both observed 72 h post-irradiation at 400X total magnification. The background stain in this and subsequent sections is propidium iodide staining for nuclear DNA.

**Figure 9:**
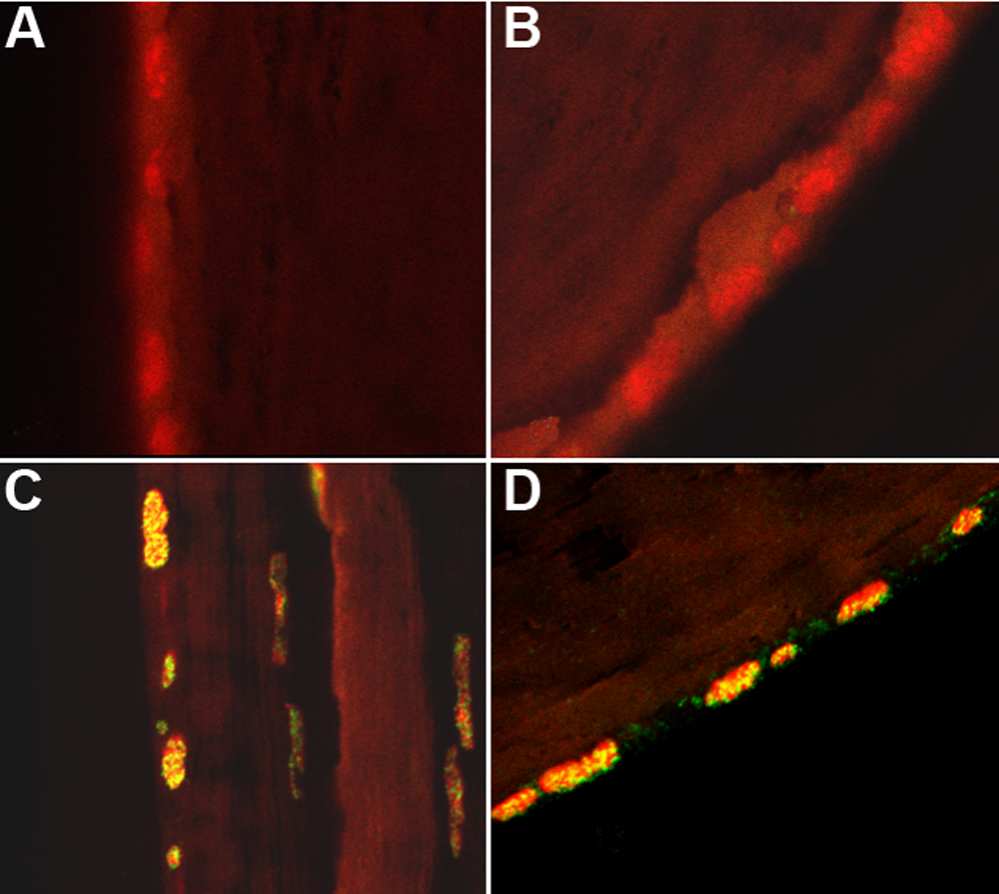
The presence of XRCC1 in the surface LEC nuclei at 72 h indicates ongoing DNA repair. Antibody for the DNA repair protein, XRCC1, was applied with a secondary fluorescent antibody and P.I. red background for nuclear DNA. The lens central zone (**A**) and near the lens equator (**B**) are from non-irradiated controls and are negative for XRCC1 expression. Panel **C** shows positive staining in the lens LEC nuclei, seen on the left. To their right is the space between lens and cornea, and at far right is the cornea. **D**: The panel shows a section from the same lens near the equator. The last two panels (**C** and **D**) are 72 h post-irradiation. Magnification 40X.

**Figure 10:**
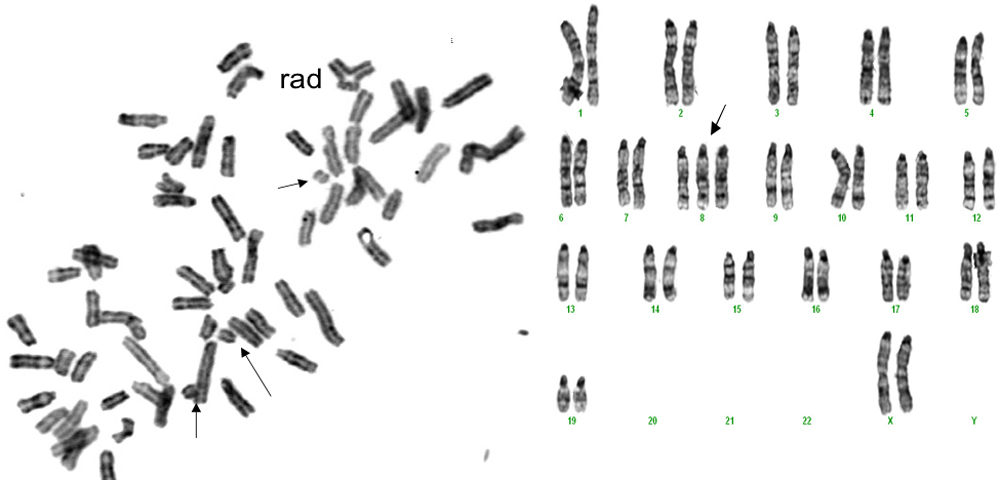
Chromosomal aberrations are present in LEC cultured for 10 days after removal 72 h post-irradiation. Left panel shows LEC from an irradiated mouse taken 72 h after irradiation and cultured for a total of 12 days in 3% O_2_, treated with colchicine for 12 h to arrest metaphases and then fixed as described in Methods, and stained with H&E. After this procedure, the metaphase spreads were read for chromosomal abnormalities. Disarray of chromosomes and broken chromosomes in the cell from the x-irradiated animal made the selection of chromosomal pairings impossible. Arrows point to broken chromosomes, and an adherence is seen at the uppermost portion in this near-tetraploid set. On the right is a spread from a same age non-irradiated mouse. One abnormality, a trisomy, is noted by the arrow. For comparisons of the damages in the several cells studied in each group see Table 1. Magnification 400X.

In addition to the LSCM studies, we prepared Carnoy’s-fixed paraffin sections in which we used an antibody to 8-hydroxyguanasine (8-OHG) to measure the in situ presence in the lens of this DNA adduct that is a result of oxidative damage [30,31,32]. This particular DNA oxidation adduct antibody, when used with a fluorescent secondary, was reliable and reproducible in our hands. Many sites for 8-OHG were present in nuclear DNA 72 h following the irradiation, which was long after the comet assay results had returned to control values. This antibody staining was absent in the controls (Figure 8). We also found large amounts of the single strand DNA repair protein, XRCC1, at nuclear sites in the nuclei of irradiated LEC at this same time and none in the non-irradiated controls (Figure 9). Metaphase spreads showed several abnormal chromosomes and chromosomal fragments. This was less common and less severe in the non-irradiated controls as shown in Table 1. An example is shown in Figure 10 (however, note one chromosomal trisomy in the control). The small number of complete metaphase spreads present did not allow a statistical comparison (See Table 1). While it appears that greater damage was present in the metaphases from the previously irradiated mice, this system will need additional studies with methods that provide increased numbers of full chromosome complement metaphase spreads.

Finally, we prepared clonal cultures separately for the irradiated mice and their non-irradiated control group in the manner noted in the Methods section. This separation measured relative capacity of individual cells from each of the two groups to replicate by producing clones with a larger or smaller number of daughter cells, i.e., to proceed through repeated cell divisions in a specific time period. However, we found neither significant clone size differences nor differences in the total number of clones formed between the irradiated and non-irradiated group (results not shown), thus LEC replication capacity was not affected in the cells surviving the irradiation event.

## Discussion

In our previous work, we have shown that the gradual development of age-related cataract (ARC) is characteristic for several strains of untreated mice and rats [33] with the advanced or mature forms (3–4+ rating on a 0–4 point scale) present in most of these animals in old age as determined by slit lamp [28,29,33]. ARC thus appeared as a universal finding for the commonly used rodent strains examined. These age-related cataracts develop slowly in pigmented mice and rats over many months beginning with mild lens opacities present in some animals as early as 12 months of age and proceeding progressively to more advanced forms by 24 months, then reaching the complete opacity of mature cataracts beyond 30 months in many of the animals [33-35]. This slow development with advancing age and population-wide predominance in the rodent condition is similar to that in humans [26]. In as yet unpublished studies, we have so far found a great similarity in the cellular and oxidative events in ARC development among the fluorescent dye, LSCM examined, age-related mouse, rat, dog, and human cortical and mixed cortical-nuclear cataracts.

In the present study, we describe in head-only x-irradiated mice a delay of several months for cataract development but then within a 30-day period the rapid appearance of cataracts that present with the same physical characteristics as those of ARC with aberrantly migrating and incompletely differentiating LEC, massive ROS presence, and evidence of retained DNA damage. After the x-irradiation, the repair of DNA strand breaks and the appearance of alkaline sensitive sites occurred within 30 min in the surviving LEC as measured by comet assay. However, these cells and possibly their immediate descendants showed in vivo the retained nuclear presence of the DNA adduct, 8-hydroxyguanasine (8-OHG), 72 h later. This was accompanied at this time by attempted or ongoing repair by the single strand DNA repair protein, XRCC1. However, the clonal measurement of in vitro replication potential in this population of LEC was not different from that of non-irradiated control donors two weeks after irradiation, although more of the cells from the irradiated population had abnormalities in chromosomal content. Thus, the initial, nonlethal, radiation-induced oxidative damage is retained in the DNA of successive proliferating LEC descendants, affecting their DNA status and causing abnormal cellular behavior, which results in cataracts months later as seen by both slit lamp and confocal microscope/fluorescent dye viewing.

Worgel [12] reported a 50-day delay in the final stage of X-ray produced cataract in rabbits with its gradual development and then a sudden change from a 3+ to the final 4+ stage within a 48 h period. A study in rats also reported several months delay in cataract appearance, but it is not clear how sudden were the final changes [36]. In general, the time to advanced cataract presence varied from two to four months depending upon animal species, age, and the dose and energy of the X-rays in these studies [12,36]. As we monitored our mice by slit lamp at 30 day intervals, we can only state that only after five or more months and then within 30 days, the opacities moved from a stage 1 or 2+ to the complete opacity of stage 3.5–4+. Indeed, the delay of several months before the irradiated lenses changed rapidly from a status to be expected at the animal’s age to complete lens opacity suggests a possible clue to their causal mechanisms.

We provide evidence that the X-ray induced oxidative damage manifests itself in progenitor LEC that repair DNA single strand breaks and related alkali sensitive sites [16] within 30 min and survive but then produce functionally defective daughter cells still capable of replication. This suggests a rapid activation of DNA repair proteins in the time immediately following the x-irradiation but incomplete or incorrect repair single base deletions or substitutions and oxidized DNA sites, resulting an incomplete or abnormal message. Loss of some LEC by apoptosis during the 5-30 min time period after irradiation is possible, although large scale apoptosis normally requires more than 30 min to take place [37,38]. These “repaired” irradiated LEC bear DNA alterations that include 8-OHG, an oxidative adduct, and show continued DNA repair activity at least 72 h after irradiation by at least one DNA repair protein, XRCC1. The single strand repair instituted by XRCC1 suggests ongoing repair and maintenance of otherwise potentially lethal retained DNA mutations that cause or threaten DNA break points [39-43]. This molecular scaffold repair protein is also required for the activity of DNA polymerase beta and its related single strand repair activity [39-42]. Although the initial DNA breakage assessed by comet assay is quickly repaired (Figure 4 and [32,43,44-46]) and LEC replication capacity was not reduced when tested at 14 days post-irradiation, we show that their later behavior in situ in the living animal was abnormal. It is well reported that several cell types including LEC with damaged and improperly repaired DNA strands may continue to replicate but continue to show evidence of retained DNA damage as seen by others [2,19,27,47-51]. This status is shown in Figure 6 and its effects in Figure 7. Whether such radiation-damaged but replication-capable LEC are responsible for the subsequent in vivo failure of LEC to proceed through normal differentiation, migration, and exclusion of ROS is not conclusively proven here. However, the lens changes that we describe here closely mimic the hallmarks of ARC in the lenses of non-irradiated old mice and rats [28,29], and the severity of ARC in both species correlates with the amount of LEC DNA derangement, their abnormal migration pattern, and the amount of oxidative damage as indicated by ROS presence [11,22,24,27-29].

Overall, our findings suggest to us that oxidative damage caused by soft X-rays represents a premature version of the status that develops in age-related cataract and that both are caused by oxidative damage to LEC DNA. In relation to this, we note that in mutant mice in which oxidative damage is reduced or slowed, the development of ARC is significantly less than that those in the same age controls [22,36] while the knockout of some antioxidant genes or otherwise increased susceptibility to oxidative damage increases the rate of cataractogenesis [22,27,52]. Thus, oxidative damage to LEC DNA, whether accruing with advanced age in non-irradiated lenses [26,28,29,53] or quick-started by a young adult exposure to soft X-rays, would seem to play a major role in the development of cataracts at least in mice and rats. We interpret the long delay in the development of the radiation-induced cataracts followed by their rapid appearance as due to a survival of LEC bearing oxidative damage that are capable of the replication seen in our clonal and metaphase spread studies but function abnormally in vivo. The subject of retained, radiation-inflicted DNA damage that results in genomic instability that remains in the descendants of the initially irradiated cells and that includes transmitted chromosomal rearrangements and genetic mutations has been addressed in the reviews by Morgan [54,55].

Finally, the progression to advanced cataracts some months post-irradiation in the studies of Worgel et al. [12] and Kodama [56] as well as in our study in which advanced cataracts develop rapidly but only after a time lag needs to be further explained mechanistically. The rapid development of the late appearing X-ray induced cataracts in our studies would necessarily result from an endpoint of rapid change in lens proteins. The changes in the lens interior have been suggested to be due to ion concentration such as calcium flux [57] as well as other molecular changes involving the lens crystallins [58,59,60]. Thus, alterations in the lens proteins that result in their aggregation are expected to be the proximate cause of the appearance of the radiation-induced cataracts. We propose that LEC with an abnormal DNA message continue to reproduce in the lens until critical numbers of cells with initial and accumulated oxidative damage result in abnormal LEC behavior including aberrant sites of migration away from the lens surface as shown in Figure 7 and seen with LSCM in our cataractous lenses by five months post-irradiation. The work of Yao et al. [61] suggests that the migration defect may involve interactions between integrin beta-1, TGF-β2, and focal adhesion kinase. Thus, failure of any message or gene phosphorylation in this pathway due to the initial radiation damage that is still propagated in the descendant LEC could be involved in the abnormal cell behavior. We propose that this reaches a crisis point when O_2_ entry into the lens at surface sites denuded of LEC as shown in Figure 7 alters the normal anaerobic status of the lens and produces ROS at a level beyond its control by antioxidant enzymes [62,23,52,22,63] with consequent lens protein coagulation and the rapid development of cataract. We recognize that this proposed explanation will require further evidence in future studies. It will be of interest to determine whether procedures such as caloric restriction [33] and an elevated expression of antioxidants [11,22,52] that significantly delay the development of ARC would also reduce the markers of LEC DNA damage, decrease the loss of LEC from the lens surface and accompanying ROS presence, and delay the appearance of soft X-ray induced cataracts. The present findings should encourage future studies on retained DNA damage in LEC and whether it is passed from damaged precursor cells to their descendants. This is an important concept in understanding the mechanisms of cataractogenesis and the importance of retention and transmission of DNA damage to daughter cells.
